# Regulation of the blood-brain barrier function by peripheral cues in health and disease

**DOI:** 10.1007/s11011-024-01468-8

**Published:** 2024-12-13

**Authors:** Kavi Devraj, Onkar Kulkarni, Stefan Liebner

**Affiliations:** 1 Department of Biological Sciences, Birla Institute of Technology & Science, Pilani, Hyderabad, 500078 Telangana India; 2 Metabolic Disorders and Neuroscience Research Laboratory, Department of Pharmacy, Birla Institute of Technology & Science, Pilani, Hyderabad, 500078 Telangana India; 3 Institute of Neurology (Edinger Institute), University Hospital, Goethe University Frankfurt, Frankfurt am Main, Germany; 4 Excellence Cluster Cardio-Pulmonary Institute (CPI), Partner Site Frankfurt, Frankfurt am Main, Germany; 5 German Center for Cardiovascular Research (DZHK), Partner Site Frankfurt/Mainz, Frankfurt, Germany

**Keywords:** Blood-brain barrier, Hormones, Chronic kidney disease, Alzheimer’s Disease, Sex-specific function

## Abstract

The blood-brain barrier (BBB) is formed by microvascular endothelial cells which are ensembled with pericytes, astrocytes, microglia and neurons in the neurovascular unit (NVU) that is crucial for neuronal function. Given that the NVU and the BBB are highly dynamic and regulated structures, their integrity is continuously challenged by intrinsic and extrinsic factors. Herein, factors from peripheral organs such as gonadal and adrenal hormones may influence vascular function also in CNS endothelial cells in a sex- and age-dependent manner. The communication between the periphery and the CNS likely takes place in specific areas of the brain among which the circumventricular organs have a central position due to their neurosensory or neurosecretory function, owing to physiologically leaky blood vessels. In acute and chronic pathological conditions like liver, kidney, pulmonary disease, toxins and metabolites are generated that reach the brain via the circulation and may directly or indirectly affect BBB functionality via the activation of the immunes system. For example, chronic kidney disease (CKD) currently affects more than 840 million people worldwide and is likely to increase along with western world comorbidities of the cardio-vascular system in continuously ageing societies. Toxins leading to the uremic syndrome, may further lead to neurological complications such as cognitive impairment and uremic encephalopathy. Here we summarize the effects of hormones, toxins and inflammatory reactions on the brain vasculature, highlighting the urgent demand for mechanistically exploring the communication between the periphery and the CNS, focusing on the BBB as a last line of defense for brain protection.

## Introduction

Neurons of the central nervous system (CNS) of higher metazoan organisms require a specific micro-environment and micro-milieu that considerably differs in cellular and molecular composition from peripheral organs (Profaci et al. 2020; Benz and Liebner 2022). The brain milieu is established and maintained in all vertebrates by the blood–brain barrier (BBB) that, with the exceptions of sturgeons, sharks and skates, is formed by endothelial cells (ECs) in brain capillaries (Bundgaard and Abbott 2008; Abbott and Friedman 2012; Profaci et al. 2020). Brain microvascular endothelial cells (BMECs) form complex and continuous, inter-endothelial tight junctions (TJs), mainly consisting of the TJ-protein claudin-5 (Cldn5) and occluding (Occl) as transmembrane proteins, thereby controlling the paracellular passage of molecules (Cong and Kong 2020). Because of their barrier function, BMECs lack fenestrae and intense vesicular traffic and therefore pronounced transcytosis, rendering the BBB endothelium a tight barrier for most polar, water-soluble molecules larger than 450Da (Abbott and Friedman 2012). However, the brain relies mainly on glucose as energy carrier that cannot cross the BBB via the paracellular route. Consequently, BMECs express specific enzymes and transporters, allowing the transport of water-soluble compounds and thereby the nourishment of the brain (for review see (Benz and Liebner 2022).

Beside TJs, brain ECs, like ECs of all other blood vessels, are endowed with adherens junctions (AJs), formed by vascular endothelial cadherin (Cdh5, cadherin-5, VE-cadherin, CD144) connected via β- or γ-catenin as well as α-catenin to the actin cytoskeleton (Giannotta et al. 2013)). Endothelial AJs are considered signaling platforms, participating in barrier regulation particularly in BMECs. Prominently, β-catenin has been identified as a crucial component of the canonical Wnt signaling pathway, that has been proven to be the main inducer of barrier properties in BMECs (Ben-Zvi and Liebner 2021). In brief, the ligands Wnt7a and Wnt7b, as well as the non-Wnt-related ligand norrin, can specifically activate the pathway in BMECs, depending on the presence of specific receptor complexes (Chang et al. 2017; Cho et al. 2017; America et al. 2022). Herein G-protein coupled receptor 124 (Gpr124; ADGRA2), reversion-inducing-cysteine-rich protein with kazal motifs (Reck) and tetraspannin 12 (Tspan12) mediate specificity for Wnt7 and norrin ligands, respectively (for a review on BBB development see (Ben‐Zvi and Liebner 2021).

Although the barrier proper is confined to BMECs, the mature differentiation of brain ECs towards the BBB phenotype is linked to their morphological connection to pericytes (PCs), astrocytes (ACs), microglia (MGs) and nerve endings, which together are denominated as the neuro-vascular unit (NVU) (Engelhardt and Sorokin 2009; Daneman and Prat 2015). More recently perivascular fibroblasts (FBs) were also shown to regulate barrier function at the NVU (Xu et al. 2022). The complex cellular structure of the NVU is typically established during embryonic and early postnatal development and requires the precise and timely interaction of cellular and molecular components and cues.

The cellular components of the NVU are not distributed equally throughout the vascular network of the CNS. As in the periphery, vessels in the CNS show a hierarchical organization in arteries, arterioles, capillaries, venules and veins. The prototype of the BBB and the NVU is realized at the level of the capillaries, which are with 5–10 μm the smallest vessels that are only covered by high numbers of PCs and by AC end feet (Abbott et al. 2010). PCs and ECs share the endothelial basement membrane (BM), whereas between PCs and ACs the extracellular matrix (ECM) is defined as the parenchymal BM (Xu et al. 2018). Although morphologically indistinguishable, the endothelial and parenchymal BM exhibit slightly different molecular composition particularly of laminins, as ACs express laminin α2, whereas ECs express laminin α4 and α5 (for review see (Morris et al. 2014).

Other cell types such as fibroblasts and smooth muscle cells are located at arterioles and, at lower numbers, at venules (Lendahl et al. 2022). Although in principle BBB functionality is present at different sections of the vascular tree, slight physiological differences in BBB characteristics have been recognized between arterioles, capillaries and venules. For example, the omega-3 fatty acid transporter Mfsd2a is predominantly expressed in venules with lower levels in arterioles, correlating in the latter with caveolae formation and neuro-vascular coupling (Kaplan et al. 2020). Aiming to understand molecular pathways involved in section-specific regulation of endothelial barrier properties is a subject matter of current research.

Clearly, the cellular interactions at the vascular wall play a crucial role for BBB characteristics. Astrocytes have been studied for decades regarding their influence on endothelial barrier properties. Particularly, in vitro co-culture systems have shown that ACs have positive effects on the transendothelial electrical resistance (TEER), reducing at the same time tracer permeability in these cultures (Arthur et al. 1987; Haseloff et al. 2005). More recently, ACs were shown to provide Wnt growth factors to the adult CNS vasculature, thereby taking part in BBB maintenance (Guérit et al. 2021). Naturally, ACs were studied in numerous pathological conditions of the CNS and have been shown to act and react in multiple ways as reactive ACs. There are excellent review articles on this topic to which we refer for more details (Siracusa et al. 2019; Brandebura et al. 2023).

Due to their close apposition to the endothelium, pericytes have been considered and shown to play a vital role in BBB formation, modulating endothelial barrier function (Armulik et al. 2010; Daneman et al. 2010). Moreover, PCs secrete the ECM component vitronectin, preventing endothelial transcytosis via integrin α5 on ECs, whereas PC-secreted laminin interacts with the dystrophin–glycoprotein complex in ACs, regulating end feet polarization (van Splunder et al. 2024). These findings imply that PCs might show various levels of dysfunction under pathological conditions when BBB function is impaired. Indeed, diminished PC numbers have been reported in aged mice, correlating with reduced PC remodeling capabilities and consequently, PCs have been considered as an early biomarker and have been implicated in the progression of Alzheimer’s Disease (AD) (Nation et al. 2019; Lendahl et al. 2019). Specifically, in carriers of the ε4 allele of apolipoprotein E (APOE4), the loss of PCs coinciding with increased levels of soluble platelet-derived growth factor β receptor (Pdgfbr) was proposed to be an early prognostic marker of disease onset independent of amyloid β (Aβ) deposition (Sweeney et al. 2018, 2019; Montagne et al. 2020; Barisano et al. 2022).

Beside AD, which is a prominent example for PC dysfunction, PC-related vascular defects have been reported in multiple CNS diseases, including Parkinson’s disease, vascular dementia, stroke, diabetic retinopathy, glaucoma, and intracranial vascular malformations (for review see (van Splunder et al. 2024). Moreover, mediators from diseased peripheral organs likely influence PC function, thereby leading to BBB damage (Hirunpattarasilp et al. 2019). Interestingly, in the human brain, two types of pericytes have been identified that are distinguished by solute transport and ECM organization, respectively, likely fulfilling selective roles within the vascular tree in health and disease (Yang et al. 2022).

In parallel to a section-specific regulation of BBB properties within the vascular tree, increasing evidence points to considerable region-specific variations of BBB signature genes (Wilhelm et al. 2016; Noumbissi et al. 2018; Winkler et al. 2022; Yang et al. 2022). Indeed, not all brain regions equally promote the formation of a BBB. In particular, areas that permit the exchange of compounds between the periphery and the brain do not possess an endothelial barrier (Kiecker 2017; Liebner et al. 2018). These regions, comprise the circumventricular organs (CVOs) and the choroid plexus which are discussed in more detail below.

Given the importance of the BBB for homeostasis of the brain parenchyma and hence for neuronal function, barrier disturbance is typically involved, or even causative for most diseases of the CNS (Liebner et al. 2018). However, not only diseases of the CNS can influence brain function, also diseases of peripheral organs may lead to substantial impairments of the BBB and consequently, of neuronal and brain function and ultimately, of behavior (Galea 2021).

Recently, the importance of the gut/intestinal-brain axis has been highlighted, suggesting a previously unrecognized, extensive communication between peripheral organs and the brain, involving bacteria-derived metabolites as well as exosomes of various cellular origin (Martin et al. 2018; Parker et al. 2019; Saeedi et al. 2019).

Regarding inter-organ communication, hormones are predestined for inter-organ communication and are known to regulate homeostatic function of multicellular organisms. Hormones can be brain-intrinsic, like neuroactive steroids (NAS), brain-derived such as adrenocorticotropic hormone (ACTH) and growth hormone (GH), or peripheral organ-derived such as adrenalin, estrogen, progesterone and testosterone/dihydrotestosterone (DHT).

Although ample evidence points to a role of steroid hormones for brain function, influencing sex-specific behavior, little is known about their specific influence on brain vasculature development along with BBB formation (for review see (Collignon et al. 2024). Current data support the view that prenatal estrogen via the estrogen receptor α (ER α) has vascular-protective function by regulating nitric oxide (NO) production in the vasculature, whereas testosterone, acting potentially via endothelial androgen receptor (AR), likely has detrimental effects on vascular function (Collignon et al. 2024). However, a deep understanding of the specific function of sex hormones at the BBB in the adult and their potential involvement in sex- and age-related risks for disease formation is missing. Nevertheless, the prevalence of stroke and AD considerably differs between men and women (Gong et al. 2023).

Regarding the influence of peripheral diseases on the BBB, it is well known for acute and chronic liver disease to induce hepatic encephalopathy, as well as for chronic kidney disease (CKD) likely to increase the risk for stroke, cognitive dysfunction, dementia and neuro-degeneration (Arnold et al. 2016; Claeys et al. 2021). In other peripheral diseases, the impact of chronic obstructive pulmonary disease (COPD), gut microbial disorders, myocardial infarction and diabetes on CNS health and neurovascular function has been investigated (Drebit et al. 1988; Braniste et al. 2014; Chan et al. 2019; Tang et al. 2020; Wątroba et al. 2023).

This review article focusses on the influence of peripheral organ-derived mediators like sex hormones and other factors on the brain vasculature and its function. We summarize the current knowledge on molecular mechanisms of endothelial barrier function, with regard to disease and sex-specific BBB disturbance. Moreover, this article attempts to highlight options to study the BBB in model systems both in vitro and in vivo in peripheral disease conditions.

## Communication between the brain and peripheral organs

The interaction of the brain with the organisms’ peripheral organs take place via nerves and the vasculature. The BBB however, is not only a unidirectional barrier preventing the entry of undesired compounds into the brain parenchyma, but also the exit of brain metabolites into the vasculature lumen. Movement of peripheral and brain-derived metabolites in and out of the brain is accomplished by various mechanisms among which passive and active transport, as well as the so-called glymphatic system contribute to (for review see (Carstens et al. 2024). To promote humoral communication with peripheral organs via the blood stream, specialized sites in the brain have developed in which vessels do not possess BBB characteristics. These so-called circumventricular organs (CVOs) confer bidirectional exchange of compounds, such as ions, signaling molecules and hormones. The CVOs are small specialized midline structures localized around the third and the fourth ventricle of the brain, comprising a group of structures with similar features but different origin (Kiecker 2017). According to their function, the CVOs either sense molecules from the blood (sensory CVOs) or facilitate the secretion of molecular cues (secretory CVOs). While the vascular organ of the lamina terminalis (VOLT; or organum vasculosum of the lamina terminalis, OVLT), the subfornical organ (SFO) and the area postrema (AP) are classified as sensory CVOs, the median eminence (ME), the neurohypophysis (posterior pituitary, PP), the pineal gland (PI) and the subcommissural organ (SCO) are determined as secretory CVOs (Benz and Liebner 2022). CVOs control crucial homeostatic functions of the organism such as thirst and water resorption in the kidney via the release of vasopressin (antidiuretic hormone, ADH) from the pituitary, as well as adrenocorticotropic hormone (ACTH) that modulates the renin-angiotensin-aldosterone system (Bichet 2018; Triebel and Castrop 2024).

Moreover, peripheral peptide hormones such as leptin and ghrelin access the brain through leaky vessels at CVOs, or via their specific transporters, hormone secretagogue receptor (GHSR) and leptin receptor (LepR), respectively at other sites of the brain, regulating hunger and food intake (Rhea et al. 2018; Spiezio et al. 2018; Jeong et al. 2021).

To achieve this communication, most CVO vessels are highly fenestrated and lack the prototypic cellular organization of the NVU, including astrocytic end feet as well as firmly attached pericytes. To protect the brain parenchyma, specialized ependymal cells, so-called tanycytes, ensheath the CVO with tight junction-forming branches (Dali et al. 2023). Given that compound exchange in the CVOs is bidirectional, it is tempting to speculate that dysregulation and diseases in peripheral organs may elicit effects in the CNS via these sites in the brain.

## Hormonal BBB regulation in health and disease

In the cardiovascular system, hormones, and particular gonadal steroids, have been described to have beneficial as well as detrimental effects, dependent on the specific hormone, sex and age (Collignon et al. 2024). Overall, female gonadal steroids like progesterone and estrogen are considered protective for the cardiovascular system, whereas male gonadal steroids like testosterone and DHT appear to have detrimental effects. However, current data are controversial regarding the effects of testosterone, as the signaling via the androgen receptor (AR) is considered to have protective function and the reduction of testosterone during aging is often associated with cardiovascular disease (CVD), metabolic syndrome (MetS), stroke, atherosclerosis and insulin resistance (for review see (Lopes et al. 2012). In general, vascular aging has been implicated in the etiology of several age-related mental disorders including AD, but mechanistic insight is still lacking (Pike et al. 2009).

So far, AR signaling as well as estrogen receptor α (ERα) has been shown to regulate several aspects of endothelial function, such as the production of nitric oxide (NO) (Goglia et al. 2010), and regulation of the vasoconstrictor endothelin-1 (ET-1) (Lopes et al. 2012). The latter was shown to be up-regulated in human AD specimen, leading to further disease progression (Palmer et al. 2012). Moreover, testosterone/DHT signaling has been implicated in regulating the endothelial inflammatory status via positive as well as negative modulation of pro-inflammatory surface receptors such as the vascular cell adhesion molecule 1 (VCAM-1, CD106) (Mukherjee et al. 2002; Death et al. 2004). These partially contradictory results might relate to the fact that testosterone and DHT can be metabolized to 17β-estradiol and 5α-androstane-3β,17β-diol (3β-diol), respectively and may therefore act via the estrogen receptor pathway (Zuloaga et al. 2012). With regard to BBB endothelial cells, little is known about the specific function of testosterone/DHT-AR signaling. However, circumstantial evidence exists that AR takes part in barrier regulation, describing AR regulation of the tight junction protein claudin-3 in Sertoli cells of the blood-testis barrier (BTB) (Meng et al. 2005). Along this line, it has been demonstrated that β-catenin, together with hypoxia-inducible factor 1α (HIF-1α), can directly interact with AR, thereby transactivating genes by binding to the androgen response element (ARE) in androgen-responsive prostate cancer cells, presenting a first hint that the AR and Wnt/β-catenin pathways can interact (Mitani et al. 2012). If such crosstalk, however, can also occur in ECs of the BBB, and whether AR-β-catenin pathway interaction may account for some of the above-described features of the aging BBB is still an open question.

Regarding non-gonadal hormones, the role of corticosteroids is well studied in several peripheral diseases including chronic kidney, liver or pulmonary disease. From the neurovascular standpoint, the corticosteroids such as hydrocortisone have been shown to improve BBB function both in vitro and in vivo. Their function appears to be mediated by mineral corticoid receptor (MR) however, the precise mechanisms are still unclear (Wijk et al. 2019).

As mentioned above, brain-intrinsic neuroactive steroids (NAS) constitute another group of molecules that are synthesized by brain cells and that modulate neuronal excitability. One of the most studied NAS is allopregnanolone that is a chemical derivative of progesterone and acts as an inhibitory allosteric modulator of the GABA_A _receptor and is therapeutically used for the treatment of post-partum depression (Maguire and Mennerick 2024). Other NAS, like members of the pregnanes, the androstanes and cholestane exhibit excitatory characteristics, acting for example as activating modulators of the N-Metyl D-Aspartate (NMDA) receptor (Paul et al. 2013b). Consequently, both pregnane (allopregnanolone and allotetrahydrodeoxycorticosterone) and androstane NAS (androstanediol and etiocholanone) have been shown to be regulators of seizure susceptibility, anxiety, and stress (Reddy 2010). The effects of testosterone, estradiol and have been elaborated for their effects on neurovasculature and BBB function as described in the previous section. However, the effects of other NAS such as allopregnanolone and etiocholanone are yet to be studied. As NAS affect seizure susceptibility, it is crucial to understand their effects on BBB functionality as one of the major hypotheses of epileptogenesis involves BBB disruption followed by leakage of excitotoxic molecules from blood-to-brain (reviewed in (Kiani 2023; Reiss et al. 2023).

### Hormonal and metabolic fluctuation and their potential effects on BBB function

#### Pregnancy

Beside the monthly menstrual cycle in human females that involves the subsequent increase of estradiol and progesterone in the follicular and luteal phase, respectively, pregnancy requires the most dramatic and long-lasting changes in the hormonal status. Importantly, estrogen and progesterone levels increase by about two-fold during the first trimester of gestation and have crucial function in blood flow regulation in the uterus and placenta (Albrecht and Pepe 2010). While progesterone levels decline in the second and third trimester, estrogen levels remain elevated. The increased levels of estrogen in the first trimester together with the human chorionic gonadotropin hormone (hCG) are likely responsible for the nausea sensation of pregnant women. Interestingly, this sensation might be detected in the *area postrema*, also known as vomiting center, that is one of the sensory CVOs and expresses high levels of estrogen receptor α (ERα) in female rats (Zhang and Hamada 2013). Given that at least via the regulation of NO, estrogens can regulate vascular tone and function, elevated levels of estrogen may also positively influence BBB functionality during pregnancy, thereby supporting a healthy state of women during this otherwise vulnerable phase (Noyola-Martínez et al. 2019).

Another interesting hormone that is roughly two- to four-fold upregulated during pregnancy is cortisol, which is a corticosteroid stress hormone released by the adrenal gland. Corticosteroids and their analogs such as dexamethasone are well-known promoter of endothelial barrier function, and therefore may also help to protect the brain vasculature from harmful barrier impairment (Jung et al. 2011; Castro-Quintas et al. 2024).

#### Testicular and prostate cancer

In young men, testicular cancer (TC) is the most common malignancy with almost 20,000 new diagnoses in Europe each year. Beside considerably improved survival rates, patients suffer from long-term treatment side effects, including decreased fertility, pulmonary toxicity, nephrotoxicity, neurotoxicity and psychosocial problems (for review see (Kirby 2020). These complications are associated with testosterone deficiency (TD), resulting from cancer- and chemotherapy-related damage to testis cells as well as from orchiectomy. In addition to TD, survivors of TC frequently develop metabolic syndrome (MetS), going along with obesity, diabetes mellitus, high blood pressure and ultimately, with an increased risk for developing cardio-vascular disease (CVD). Together these health complications may influence neuronal function, leading to psychological problems. MetS has already been shown to have considerable negative effects on the BBB, given that patients suffering from MetS have a systemic pro-inflammatory phenotype that leads to junctional weakening and transmigration of inflammatory cells (Mauro et al. 2015; Dyken and Lacoste 2018; Sheikh et al. 2021). If TD and MetS also have additive disturbing effects on the BBB is currently not explored.

#### Menopause, breast and ovarian cancer

Estrogens, decline dramatically during menopause, and hormone replacement therapy (HRT) is considered as a preventive therapy against vasomotor symptoms such as insomnia and hot flushes, osteoporosis, psychological complications as well as cognitive impairment and Alzheimer’s disease (AD) (Alblooshi et al. 2023). Given that in particular the CNS-related diseases coincide with BBB defects, it is tempting to speculated, that the dramatic and persisting hormonal decline during menopause directly leads to diminished barrier function, thereby promoting dementia. A potential link between estrogens and BBB function might be the downstream regulation of annexin A1 (ANAXA1) that is an anti-inflammatory protein, at least secondarily effecting endothelial barrier properties (Herson and Kulkarni 2022). Moreover, estrogens regulate serotonin receptors and thereby likely contribute to diminished serotonin signaling, resulting in an increased risk for developing depression in menopausal and post-menopausal women (Herson and Kulkarni 2022). Interestingly, a direct link between depression and BBB function has been recently shown, suggesting that diminished BBB function promotes depression, while sealing the BBB can ameliorate depressive symptoms (Menard et al. 2017; Dion-Albert et al. 2022).

Along with menopausal estrogen decline, women show an increased risk of developing dementia and AD. Although a direct connection of estrogens to amyloid β (Aβ) formation and processing is still controversial, there is circumstantial evidence that estrogen, via ERα but not ERβ, is effective in reducing Aβ load (Anastasio 2013). ERα might achieve this by promoting the non-amyloidogenic processing of amyloid precursor protein (App) through the activation of the mitogen-activated protein kinase (MAPK)/extracellular-signal-regulated kinase (ERK) pathway, and the downstream activation of a disintegrin and metalloprotease domain 17 (ADAM17, an α-secretase candidate) (Shi et al. 2014). Interestingly, the ε4 allele of APOE4 is the predominant genetic risk factor for late-onset AD, and the APOE4-related risk of developing AD is greater in women than in men (Valencia-Olvera et al. 2023). There is evidence from preclinical models that female homozygous carrier of the APOE4 allele benefit less from the memory-enhancing effect of 17β-estradiol via the ERα receptor (Taxier et al. 2022). These effects may however not be related to major differences in RNA expression, evidenced by bulk RNA-Seq data (Liu et al. 2021).

Interestingly, not only the physiological decline in hormones during menopause, but also HRT may, under certain circumstances, result in an increased risk of developing dementia (Rossouw et al. 2002; Shumaker et al. 2003). This apparent controversy clearly demonstrates that more research is desperately needed to understand and to therapeutically exploit the hormonal system.

## Peripheral diseases and BBB dysfunction

### Chronic kidney disease (CKD)

Chronic kidney disease (CKD) is a chronic, progressive disorder that leads to low glomerular filtration rate (GFR), requiring hemodialysis and kidney transplantation in later stages. CKD also imposes secondary complications such as hypertension, anemia, metabolic acidosis, bone disease and neurological complications (Chillon et al. 2016; Collaboration et al. 2020). End stage disorders like CKD potentially affect the nervous system at various levels from the central nervous system (CNS) to the peripheral nervous system (PNS) (Chillon et al. 2016; Puy et al. 2018).

Cognitive dysfunction and dementia have high prevalence (about 70% of individuals undergoing hemodialysis) in CKD patients. Decreasing GFR contributes to enhanced levels of uremic toxins such as urea, creatinine, guanidine, uric acid, homocysteine along with a chronic inflammatory condition leading to increased cytokine (IL-1β, IL 6, IL 10 and TNF-α) levels. Cumulatively, this contributes to endothelial dysfunction and disruption of the BBB, exposing the CNS to direct neuronal toxicity (Bjerring et al. 2012; Bugnicourt et al. 2013; Levin et al. 2017; Carney 2020). Uremic toxins produced in CKD can pass through the BBB and cause cognitive dysfunction and neurodegeneration (Sun et al. 2020). Moreover, uremic toxins such as phosphate, para-cresyl sulfate (PCS), indoxyl sulfate (IS), and fibroblast growth factor 23 (FGF23) have been reported to increase the risks of cognitive impairment in patients with CKD (Yeh et al. 2016; Adesso et al. 2017, 2018; Karbowska et al. 2020).

CKD pathogenesis of associated with cognitive impairment (CI) involves vascular and non-vascular mechanisms (Drew et al. 2019). BBB dysfunction and microbleeds were shown to corelate with CI in patients with CKD (Bjerring et al. 2012; Drew and Weiner 2014; Six et al. 2020). The impairment of blood flow autoregulation and loss of BBB integrity due to systemic inflammation, vascular changes such as calcification, and accumulation of uremic toxin leads to CI in CKD (Drew and Weiner 2014; Karasavvidou et al. 2018; Six et al. 2020). The European Uremic Toxins Work Group (EUTox) suggests that uremic toxins accumulate in the context of renal dysfunction, which in turn either causes neuroinflammation via direct activation of microglial cells and neurotoxicity or indirectly, affecting the vasculature by the virtue of endothelial cell activation, vascular calcification or alteration of vascular permeability (Vanholder et al. 2003; Six et al. 2020). Uremic toxins are classified as protein-bound, middle or small water-soluble toxins, based on their protein-binding properties and molecular weight. Large and middle molecules and certain protein-bound toxins tend to accumulate in the systemic circulation due to their poor clearance by dialysis (Vanholder et al. 2003; Masereeuw and Verhaar 2020). In fact, patients with renal transplantation showed an improved clearance of these toxins and an improved cognitive function, suggesting an inability of current hemodialysis procedures to clear these toxins effectively from the circulation in CKD patients (Linde et al. 2020). Clearance of uremic toxins is not only dependent on glomerular filtration but also has an underestimated role of proximal tubular epithelial cells which, through the presence of various membrane transporters actively eliminate the protein bound toxins through urine (Masereeuw et al. 2014). This could in part explains the inadequate clearance of these protein-bound toxins by the dialysis procedure which primarily function by filtration mechanism.

Among the various uremic toxins IS and pCS have been shown to correlate with CI in CKD (Yeh et al. 2016). These toxins tend to accumulate in the brain, showing increased levels in the brainstem, cerebellum, striatum and hippocampus (Karbowska et al. 2020; Li et al. 2021). The attenuated expression or function of the membrane transporters like OAT3 can be responsible for these elevated levels of toxins in the brain (Ohtsuki et al. 2002). In a preclinical CKD study, a significant decrease in the RNA and protein expression of Abcg2/Bcrp, Mrp2/4, Oat3, Oatp2/3 and P-gp was found in brain endothelial cells (Naud et al. 2012). In line with this observation, rat brain ECs stimulated with rat CKD serum in vitro showed downregulation of several transporters. These studies clearly indicate an alteration in the BBB membrane transporter expression and function in CKD, which are associated with accumulation of various uremic toxins in the brain. Treatment with an oral active carbon absorbent (AST-120) reduced the IS accumulation in the frontal lobe and hippocampus and ameliorated the progression of CI in a mouse model of 5/6 nephrectomy (Sun et al. 2021). Stimulation of brain endothelial cells with urea, one of the uremic toxins, or uremic serum from CKD patients showed reduced expression of tight junction proteins like claudin-5 and disruption of the actin cytoskeleton. Similar effects of urea on the intestinal epithelial barrier structure and function have been shown in preclinical models of CKD and in vitro studies (Faucher et al. 2023). IS and pCS also have been shown to induce endothelial dysfunction in brain endothelial cells by inducing oxidative stress. IS a known agonist of the aryl hydrocarbon receptor (AHR) and induces BBB dysfunction upon binding to AHR. Interestingly, AhR^−/− ^knockout mice overloaded with IS in the drinking water are protected from BBB disruption and subsequent cognitive impairment (Dou et al. 2018; Bobot et al. 2020). Along this line, Ahr has previously been implicated in BBB regulation as an upstream regulator of the cytochrome P-450 isoform Cyp1b1 in cooperation with the Wnt/β-catenin pathway (Ziegler et al. 2016). However, Ahr is a highly context-dependent transcription factor, that in mouse brain microvascular endothelial cells (MBMECs) transcriptionally regulates Cyp1a1 and Cyp1b1 in the absence and presence of active β-catenin, respectively (Ziegler et al. 2016). Interestingly, Cyp1a1 has been shown to be involved in the metabolic activation of heterocyclic amines and polycyclic aromatic hydrocarbons that are substrates for the generation of indoxyl compounds such as indoxyl sulfate (Guengerich and Shimada 1991; Banoglu et al. 2001). If cytochrome P-450 enzymes are indeed involved in IS metabolism at the BBB, leading to barrier breakdown is currently not explored.

The BBB is created by the interaction between the tightly sealed BBB ECs and associated cells of the NVU like astrocytes and pericytes. IS can induce cell death, increase pro-inflammatory cytokine expression, and alter mitochondrial membrane potential in primary astrocytes (Lin et al. 2019). Quinolinic acid, another uremic toxin, can reduce glutamine synthetase activity and induce chemokine receptor expression in human astrocytes (Guillemin et al. 2003). In another report, homocysteine was shown to increase H_2_O_2 _production and to trigger apoptosis in ECs (Assem et al. 2018).

#### CKD and Immune modulations and its impact on BB

CKD has a significant impact on the immune system. Chronic kidney disease induces inflammation by increasing Toll-like receptor-4 (TLR4), cytokine and cathelicidin expression in neutrophils and monocytes (Grabulosa et al. 2018). Monocytes and neutrophils from CKD patients on dialysis showed increased expression of TLR2 and TLR4 that can likely be attributed to the increased response to stimulation of these cells (Sela et al. 2005; Gollapudi et al. 2010; Grabulosa et al. 2018). Uremic sera and toxins like IS have been shown to affect the phenomenon called ‘trained immunity’ in which innate immune cells can build up immunological memory resulting in enhanced responsiveness to subsequent stimulation. Healthy monocytes pre-exposure to CKD sera or IS have shown an enhanced response towards to the stimulation with LPS and PAM3, both are routinely used as TLR agonist (Kim et al. 2023). The observed effect is due to an increased activation of arachidonic pathway, epigenetic modifications and metabolic rewiring. In fact, monocytes isolated from patients with end-stage renal disease (ESRD) showed increased expression of proinflammatory cytokines in response to LPS when compared to the age matched heathy controls (Kim et al. 2023). On the other hand, uremia/uremic toxins (IS, pCS, pCG) have been shown to reduce the phagocytosis process and oxidative bursts in macrophages and neutrophils, respectively, as well as leukocyte-endothelial interaction, affecting vascular leakage (Vanholder et al. 1995; Mahajan et al. 2005; Lim et al. 2007; Pletinck et al. 2013).

Patients on intermittent hemodialysis exhibit lower number of myeloid as well as plasmacytoid dendritic cells. The isolated circulating precursor (pre)-DC subsets from hemodialysis patients were functionally impaired with reduced expression of co-stimulatory molecules upon LPS stimulation, and reduced IFN response upon herpes simplex virus stimulation when compared to healthy controls and patients with renal transplantation (Hesselink et al. 2005; Lim et al. 2006; Paul et al. 2013). This suggest that renal transplantation can bring the DC function to normalcy. Similar to its effect on innate immune system like we have discussed earlier, the CKD also affect the functionality of adaptive immune response. CKD has been shown to exhibit reduced numbers of lymphocytes (B and T cells), activation and depletion of naive T lymphocytes leading to lower T-cell receptor repertoire diversity (Yoon et al. 2006; Betjes et al. 2011; Huang et al. 2017).

The other immune cell species which is impacted by the uremia or CKD is the mast cell. CKD-associated pruritus (CKD-aP) is the most common skin symptom associated with uremia and appears in almost half of patients with advanced CKD (Rayner et al. 2017; Shirazian et al. 2017). The accumulation of uremic and other substances like parathyroid hormone, calcium, phosphorus, and aluminum in CKD patients has been postulated as one of the mechanisms involved in the development of CKD-aP. The assessment of metabolic profile of CKD patients with purities further identified LysoPE (20:3(5Z,8Z,11Z)/0:0), p-Cresol glucuronide, LysoPC(20:2(11Z,14Z), hypotaurine, 4-aminohippuric acid, LysoPC(16:0), phenylacetic acid, kynurenic acid and androstenedione as the biomarkers of CKD-aP (Wu et al. 2018). Progression development of CKD is also influenced by the mast cell activation and factors released by the mast cells. Tryptase and chymase, the two of important mast cells specific proteases have been shown to play an important role in the progression of CKD, and accumulation of mast cells in the renal tissue has been shown to be detrimental to kidney function (Vibhushan et al. 2020). Mast cell-induced vasodilation and increased vascular permeability contributes to infiltration of immune cells into the renal tissue, thereby further damaging the kidney. Mast cell-specific proteases like tryptase, chymase and carboxypeptidase A3 have been shown to be increased in the serum of CKD patients and to be corelated with renal function (Owens et al. 2018).

Collectively, these immune modulatory events in CKD might directly or indirectly contribute to BBB dysfunction, by modulating junctional integrity as well as endothelial expression of transporters, which has previously been proposed (Faucher et al. 2023). There is a plethora of scientific studies reporting the impact of inflammatory cytokines of BBB function and integrity. The uremic toxins have been shown to increase expression and secretion of proinflammatory cytokines by neutrophils and monocytes as well as to sensitize these cells for stimulations by pathogen-/damage-associated molecular patterns (PAMPs)/DAMPs) due to “trained immunity”. Interestingly, capillary-adhered neutrophils have been shown to negatively influence cerebral blood flow, promoting disease progression in AD and antibodies directed against neutrophils ameliorated AD-related cognitive impairments (Hernández et al. 2019). IL-6, TNF-α, IL-1β are cytokines known to cause BBB dysfunctions, and therapies targeting these cytokines or their receptors can restore BBB integrity in various disease pathologies. TNF-α, which is either produced by microglial cells or injured neurons or inflammatory macrophages can disrupt TJs and can induce necroptosis in BMECs through TNFR1 (Aslam et al. 2012; Chen et al. 2019). TNF-α can also activate brain ECs and ACs to express chemokines and cytokines thus recruiting leukocytes. Moreover, it is also a potent inducer of ICAM-1 and VCAM-1 by brain ECs (O’Carroll et al. 2015). Apart from leukocyte adhesion molecules and cytokines TNF-α can induce expression of MMP-2, MMP-3 and MMP-9, which further leads to disruption of TJs and BBB dysfunction (Ding et al. 2019).

IL1-β on the other hand, induces BMECs to express IL-6, sICAM-1, soluble TNF receptors, granulocyte-colony stimulation factor (G-CSF) and granulocyte-macrophage colony stimulation factor (GM-CSF). The effect of IL-1β on brain ECs differs to that from TNF-α (O’Carroll et al. 2015). IL-1β activates ACs to exacerbate the progression of multiple sclerosis through breakdown of the BBB (Argaw et al. 2006). Furthermore, IL-1β induces secretion of IL-6 and TNF-α and decreases TEER in an in vitro BBB model, using transfected human brain microvascular endothelial cells (Labus et al. 2014). In mice, burn injury also exhibit altered BBB function with increase in paracellular and transcytotic permeability, which is mediated by systemic levels of IL-6 and IL-1β (Yang et al. 2020).

As mentioned above, CKD also exhibits increased activation of mast cells and increased levels of tryptase and chymase in CKD serum. The cytokines IL-6, TNF-α, IL-1β are known to cause BBB dysfunctions and therapies targeting these cytokines or their receptors can restore BBB integrity in various pathologies. For example, mast cell deficient mice are protected from cognitive impairment in a 5XFAD model of Alzheimer’s disease (Lin et al. 2023). The neuropenetrance of Japanese encephalitis virus (JEV) is mediated by mast cells derived chymase. Chymase enhances JEV-induced breakdown of the BBB and cleavage of tight junction proteins, while the inhibition of chymase rescued the neurological deficits by reducing the BBB leakage and JEV neuro-penetrance (Grabulosa et al. 2018). On the other hand, tryptase also plays an important role in neuroinflammation, BBB dysfunction and cognitive impairment. But the role of mast cells and mast cell-derived factors is yet to be explored in the context of BBB dysfunction and cognitive impairment associated with CKD.

Beside the immune modulatory effects taking place in CKD patients, pronounced effects on the endocrine system have been documented (Table 1).

**Table 1:** CKD and endocrine system

Hormone	CKD Phenotype	Observations	Effect on BBB	Reference
Erythropoietin (EPO)	Reduced expression in the context of actual blood haemoglobin concentration	Reduced synthesis of endogenous erythropoietin (EPO) due to loss renal parenchyma	• EPO Protects BBB integrity	(Martínez-Estrada et al. 2003; Locatelli et al. 2013; Chu et al. 2014; Suzuki and Yamamoto 2016)
Vitamin D and metabolites				
a. 25(OH)D3 b. 1,25(OH)2D3	Reduced expression in CKD patients Reduced expression and activity in CKD	• Increased clearance by the kidney • Excess removal due to dialysis procedure • Reduced activation of 25(OH)D3 due to lower expression of 1αhydroxylase • Reduced expression of Vitamine D receptors	• Vitamine D protects BBB integrity	(Dusso 2011) (Dusso and Tokumoto 2011) (de Oliveira et al. 2020)
Follicle-stimulating hormone	Elevated expression in CKD	• Leads to increased systhesis of testosterone-binding globulin • Also contribute to the progression of renal fibrosis	Lowering FSH maintains BBB permeability	(Li et al. 2021) (Zhang et al. 2019) (Wilson et al. 2008)
Prolactin	Elevated expression in CKD	• Reduced clearance and increase secretion • Negatively corelates with cardiovascular function due to endothelial dysfunction • Reduced secretion of gonadotropin releasing hormone (GnRH) in turn reduced levels of testosterone	Prolactin protects BBB	(Huang and Molitc 2021) (Holley 2004) (Ros and Carrero 2012) (Rosas-Hernandez et al. 2013; Rosas-Hernandez et al. 2013; Rosas-Hernandez et al. 2015)
Testosterone	Reduced expression in CKD	• Low levels are attributed to Leydig cell dysfunction • Absence of gonadotropin pulses required for testosterone production • Decreased serum concentration of androstenedione and dehydroepiandrostrone	Testosterone protects BBB in the adult	(Romejko et al. 2022) (Garibotto et al. 2021) (Atallah et al. 2016)
Estrogens	Low expression in CKD	17β-estradiol is Reno protective	BBB protective effects	(Ma et al. 2021) (Rajabi et al. 2024) (Xiao et al. 2017)
Growth hormone	Elevated expression in CKD	GH resistance attributed to reduced expression of GH receptors on target organs Reduced clearance of GH in CKD	rhGH/somatropin protects BBB	(Tönshoff et al. 2005) (Haffner et al. 1994) (Zaky et al. 2021)

### Chronic obstructive pulmonary disease (COPD)

COPD is a peripheral disease known to cause vascular dysfunction. The pathobiological mechanisms of COPD has been reviewed by Chan and colleagues (Chan et al. 2019). Importantly, half of COPD patients suffer from MetS as a comorbidity. Increased obesity, hyperglycemia, hyperinsulinemia, and increased triglyceride levels were collectively reported in COPD patients. Other major comorbidities of COPD include ischemic disease and cognitive dysfunction. Hypoxia, increased neutrophil count during exacerbation of COPD, increased cytokines, total oxidative status, and acute inflammatory markers like C-reactive protein are upregulated systemically in COPD (Stanojkovic et al. 2011; Stanojkovic et al. 2013). The analysis of serum for total antioxidant status (TAS) and the prooxidant-antioxidant balance (PAB) during exacerbation of COPD showed systemic oxidative stress in patients with COPD (Stanojkovic et al. 2011; Stanojkovic et al. 2013). The increased oxidative stress leading to increased levels of reactive oxygen species (ROS) in COPD patients potentially contributes to cognitive dysfunction by an impairment of the NVU and thus of BBB function (Lahousse et al. 2015). It is further known that free radical levels increase in plasma in COPD patients (Rossman et al. 2013); Dobric et al. 2022). That ROS negatively influence BBB function has previously been shown, suggesting that also oxidative stress in the periphery may lead to BBB dysfunction (Lehner et al. 2011; Ahmad et al. 2012). The detailed mechanisms however are unexplored and therefore unclear.

### Gut microbial disorders

The impact of gut microbial disorders on microvasculature (Kiouptsi et al. 2023) and specifically on the brain microvasculature (Braniste et al. 2014; Tang et al. 2020) has recently been investigated. Braniste and colleagues demonstrated that lack of gut microbiota was associated with increased BBB permeability (Braniste et al. 2014). They could further show that germ-free mice treated with short chain fatty acids led to an improved BBB phenotype. Another gut microbial metabolite lipopolysaccharide (LPS) is well known to cause BBB disruption (Banks and Erickson 2010; Vutukuri et al. 2017). Gut microbial metabolites are involved in several complications including CNS diseases (Canfora et al. 2019; Morais et al. 2021). Interestingly, the systemic accumulation of uremic toxins is in part due to the inadequate clearance as well as due to the gut dysbiosis associated with CKD. Gut dysbiosis in CKD has been found to be responsible for over-production of certain protein-bound uremic toxins such as indoxyl sulfate (IS), p-cresyl sulfate (pCS), and indole-3-acetic acid (IAA) (Joossens et al. 2019; Glorieux et al. 2020; Rysz et al. 2021). Precisely how these gut metabolites affect neurovascular function in various neurological diseases is yet to be elaborated.

### Diabetes mellitus

Diabetes mellitus is also associated with BBB dysfunction resulting from oxidative stress due to hyperglycemia. A dysregulation of glucose transporter levels, P-glycoprotein, LRP1, amino acid transporters due to hyperglycemia also impacts BBB function due to associated metabolic changes within the brain endothelium (Drebit et al. 1988; Shikha et al. 2014; Wątroba et al. 2023). Advanced glycation end products resulting from diabetes lead to induction of VEGF that is well known to cause vascular permeability. Furthermore, diabetes is also reported to induce matrix metalloproteases (MMPs) that have been shown to cause BBB impairment by their effects on the basement membranes at the NVU (Hawkins et al. 2007).

Diabetes-associated glycemic imbalance has been reported to alter the expression profile of BBB molecules (Sakamuri et al. 2023) and also to affect cognitive changes due to dysfunction of the BBB (Wątroba et al. 2023). These studies go along with the classic studies by Simpson et al., showing altered expression of GLUT-1 in BBB microvessels from streptozotocin-induced diabetic rats (Simpson et al. 1999). In vitro studies also demonstrate BBB breakdown upon glucose and oxygen imbalance (Ahmad et al. 2012; Page et al. 2019). Insulin resistance is a key characteristic of type 2 diabetes. Even in the brain, the multiple functions of insulin, including metabolic, synaptic and immune related ones, are affected by type 2 diabetes. This insulin resistance could also be a result of poor transport of insulin across the BBB (Arnold et al. 2018; Rhea 2019). Furthermore, a host of diabetes-related neurological disorders such as stroke, AD and epilepsy have been reported, and most of them being associated with BBB dysfunction (Shikha et al. 2014; Venkat et al. 2017).

## In vitro models

Due to the complexity of the BBB, in vitro modeling of NVU cells is key for functional BBB studies. The barrier function itself is established by brain endothelial cells, and this is regulated by all cell-types of the NVU including pericytes, astrocytes, microglia, and neurons. There are several models in the field most of which employ single cell-types and primary brain microvascular endothelial cells (BMECs) (Czupalla et al. 2014; Williams-Medina et al. 2021). Given that BMECs are the primary cell-type in barrier function, a minimalistic model is a monolayer of brain endothelial cells in culture. There are several BBB models that comprise only endothelial cells, for which the cell source, species, days in vitro, extracellular matrix components, and cell growth and differentiation media components, the geometric layout of the luminal (or apical, blood-side) and abluminal (or basolateral, brain-side) have all been considered to be of importance (Czupalla et al. 2014). While there are certain general features of all BBB models such as low paracellular permeability, expression of classic BBB marker proteins including tight junction proteins, and specific transporters (GLUT-1, PgP), each model has its specific elements that is tailored for the disease or condition being studied.

Brain endothelial cells in co-culture with either astrocytes or pericytes is a BBB model frequently used for elaborating the role of NVU cells in regulating BBB function. Such co-culture models comprise both primary (bovine, porcine, rat or mouse brain sources) or immortalized brain endothelial cells (bEnd3, bEnd5 or HMEC/D3) in co-culture with primary fetal astrocytes (mouse, rat, human) or primary brain vascular pericytes (Bhalerao et al. 2020). More recently, induced pluripotent stem cells (iPSCs) have been used to obtain major NVU cell-types for in vitro BBB modeling (Workman and Svendsen 2020). These co-culture and endothelial mono-culture models have been used to mimic disease conditions as well. Ischemic stroke, brain cancers, brain infections, Alzheimer’s disease, and neuroinflammation have all been studied in vitro using tailored BBB models (Erickson et al. 2020).

There is ample evidence for BBB damage and subsequent neurological impact of peripheral diseases such as diabetes, chronic kidney disease, chronic hepatic disease, chronic obstructive pulmonary disease (COPD), and gut-microbiota disorders (Bernard et al. 2019; Parker et al. 2019). However, there are very few studies investigating these mechanisms. Furthermore, in vitro models for studying the influence of peripheral disease on BBB function are lacking. Culture of endothelial cells with peripheral cells such as skin fibroblasts or chondrocytes has been pursued from the perspective of vascularization of engineered tissues. However, their co-culture particularly using brain microvascular endothelial cells with peripheral cells in order to investigate the role of peripheral factors on BBB function has not been systematically investigated in vitro.

**Fig. 1:**
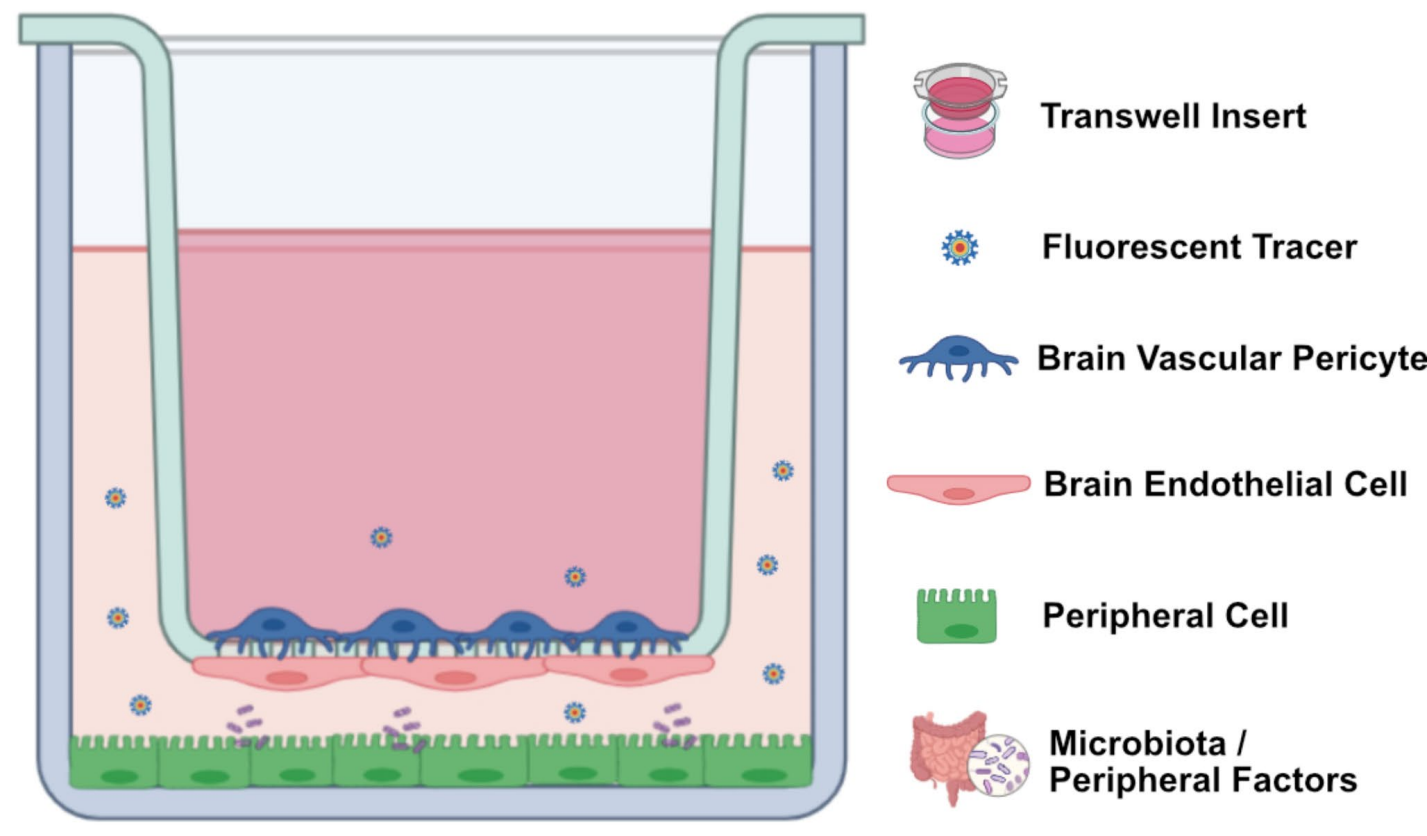
Scheme of a potential in vitro co-culture setup to study the effect of peripheral organ-derived metabolites and compounds on endothelial barrier properties. Created with BioRender.com

An in vitro BBB model comprising brain endothelial cells in non-contact co-culture with gut epithelial cells (e.g. CaCo2), kidney epithelial cells (MDCK), hepatocytes or alveolar epithelial cells could potentially be used for studying the impact of gut, kidney, liver or lung disease on BBB dysfunction in vitro (Fig. 1). The disease conditions of the gut or kidney epithelia can be mimicked by addition of serum from a preclinical animal disease model or from a patient to the basal chamber media comprising these peripheral cells. Inclusion of gut microbiota (or their metabolites) or uremic toxins, bile products or smoke extract could also mimic the peripheral disease of the above organs in vitro. The impact of such a disease scenario on BBB dysfunction can then be studied by fluorescent tracer permeability assays (as illustrated in Fig. 1), or continuous impedance measurements across brain endothelial cells (Czupalla et al. 2014; Hansen et al. 2021; Spitzer et al. 2022). Primary brain endothelial cells or their co-culture with other primary NVU cells in the top chamber could lead to a more robust modeling of BBB function in peripheral disease. In a more in vivo-like syngeneic model, the NVU cells or the peripheral cells could be differentiated from iPSCs (Workman and Svendsen 2020).

Flow is an important component for dynamic modeling of the BBB in vitro (Williams-Medina et al. 2021). The flow aspect has several advantages compared to a static 2D transwell system that has been classically used. These include a physiological release of nitric oxide based on flow pattern, in vivo-like expression profile of BBB markers based on mechano-transduction, and drug permeability profile that is affected by drug residence time in the vessels. Also, disease modeling could be more in vivo like particularly in hypoxia/ischemic conditions where flow and no flow conditions could affect the reperfusion damage. Also, immune cell and tumor cell trafficking could be affected by flow in studies of brain metastasis and neuroinflammation.

The NVU and peripheral cells could also be included into microfluidic devices incorporating physiological flow conditions in order to obtain state-of-the-art in vitro model systems that utilize fewer cells, thus enabling patient-specific modeling of the peripheral disease and its influence on BBB function (Oddo et al. 2019; Wevers and Vries 2023). These microfluidics-based BBB models allow incorporating all major cell types of the NVU that potentially affect the BBB (Spitzer et al. 2022; Spitzer et al. 2023) as the number of cells required in these models are minimal. 3D geometries are feasible in microfluidic based models, allowing more in vivo-like anatomic organization and flows.

Gut, kidney, liver or lung on chip have all been proposed (Wu et al. 2020; Koyilot et al. 2022). Thus, a combination of these chips with the BBB on chip that has also been proposed could pave the way for microfluidic devices to study the impact of peripheral disease on BBB.

## In vivo models

The neat interplay of the involved cell types at the neuro-vascular unit, which is crucial for its structure and ultimately of BBB function, has challenged the development of appropriate in vitro models. Although reductive in vitro approaches, using various mono- and co-culture settings, have great value in pharmacology and inflammation research, many aspects in mechanistic understanding of BBB regulation still require the use of in vivo models. On the one hand, genetic modulation of signaling pathways in cell type-specific and inducible manner has become a standard procedure in advanced mechanistic studies, allowing the precise manipulation of a gene within a pathway of interest at a specific timepoint and in a specific cell type. Herein, novel advanced technologies go hand-in-hand, as identification of cell-specific gene expression by single cell sequencing techniques, providing a precise atlas of cell-specific profiles, and allowing the generation of cell-specific alleles that for example can be used to drive expression of nucleases such as the Cre-recombinase of the bacteriophage λ family (for review see (Kim et al. 2018). As mammalian organisms and due to their genetic similarity to humans, mice are frequently used when it comes to disease models, for which either surgical techniques need to be applied (middle cerebral artery occlusion (MCAO) model of stroke) and/or behavioral read outs (learning and memory like in AD) are required (Li and Zhang 2021; Sanchez-Varo et al. 2022; Zeng et al. 2023; Zhong et al. 2024). Moreover, in mouse models differently sized molecular tracers can be applied intravenously and allow to visualize vascular leakage up to the cellular level (Devraj et al. 2018). Additionally, tracers can also be applied into the*cisterna magna*, which helped to identify the so-called glymphatic system, which is a hot topic in the field of CNS vasculature and barrier research (Xuan et al. 2022). Last but not least, mouse models are used to explore gene therapy approaches targeting the BBB in CNS diseases (Martin et al. 2022; Soufizadeh et al. 2024; Trevino et al. 2024). Developmental and mechanistic studies are frequently conducted in model organisms with shorter generational sequence like zebrafish. As teleosts, also zebrafish has an endothelial BBB and because of the broad genetic tool box, it is an ideal model to study brain angiogenesis and BBB development (Eliceiri et al. 2011; Vanhollebeke et al. 2015; Schevenels et al. 2024).

Although beyond the scope of this review, it should be mentioned that mouse and rodent models in general support a broad variety of techniques to image the functionality of the BBB, ranging from non-invasive techniques such as MRI, PET and CT to invasive methods such as multi-photon microscopy after cranial window surgery and comparable techniques. These options, along with the ability of genetic manipulation and the plethora of available disease models with substantial similarity to humans, makes the mouse the organism of choice for disease-related, preclinical research.

## Conclusions and perspective

**Fig. 2:**
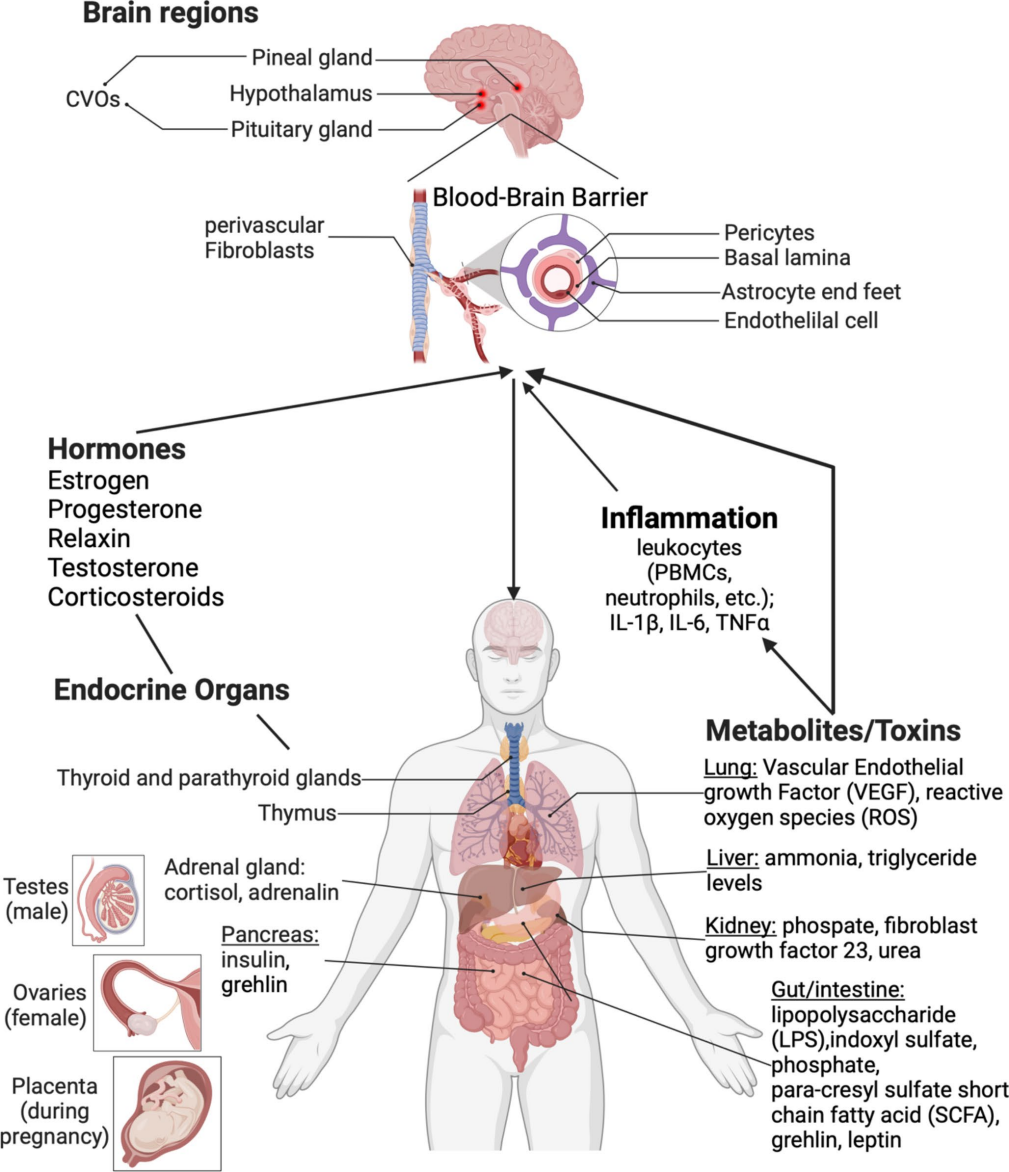
Summarizing scheme of hormonal, metabolite and toxin interaction from peripheral organs to the brain and the blood-brain barrier in particular. Created with BioRender.com

The blood-brain barrier (BBB) formed by endothelial cells (ECs) fulfills its function in conjunction with the closely attached cells of the neurovascular unit (NVU). In the past the NVU and the BBB have been considered as relatively rigid and, at least in healthy organisms, as little regulated structures. Over the last decades, accumulating evidence suggests that the BBB is a highly dynamic structure that is regulated by various body intrinsic as well as extrinsic cues (Guérit and Liebner 2017; Daneman and Engelhardt 2017). In this context, regulation of BBB function by peripheral organs and vice versa is gaining increasing attention by the scientific community. Herein hormones of different origin and function such as in the menstrual cycle, pregnancy, menopause and determination of male characteristic, which physiologically underly dramatical changes in a cyclic or sporadic manner as well as during ageing, are of particular interest. Most data indicate that female hormones derived from organs such as the uterus, ovary and the placenta have a definitive effect on the BBB (Collignon et al. 2024). However, circumstantial evidence from healthy as well as diseased humans suggest that male hormones likely participate in vascular and thereby also to BBB functionality. Given that the hormonal status of males and females has been studied in great detail, very few aspects about their role in the vascular system and at the BBB have been elucidated, leaving a great new field of research to be explored. Interestingly, several, if not all, diseases of peripheral organs, including the gonads, heart, lung, liver and kidney generate hormones as well as specific metabolites and toxins that influence various reactions in the organism comprising the inflammatory as well as hormonal system (Table1; Fig. 2). Although it does not come as a surprise that chronic and ultimately life-threatening diseases such as CKD, liver disease, COPD and others may influence BBB function, potentially contributing to neurological complications, surprisingly little is known about the mechanistic relationships. Beside blood contained cellular (intrinsic) and extrinsic toxins like LPS, that directly mediate effects on the BBB, cell-derived or bacteria-derived extracellular versicles may add another level of complexity that has poorly been explored in the past years (Ridder et al. 2014). BBB dysfunction has been shown to be associated with various neurological pathologies. However, if BBB impairment is a prerequisite for all types of CNS disease and at which step during disease progression BBB dysfunctions develop is not entirely known. Although there are recent evidences suggesting that BBB dysfunction precedes the cognitive impairment associated with myocardial infarction or Alzheimer’s disease (Montagne et al. 2015; Nation et al. 2019). Similar to these findings, peripheral traumatic injuries like burn and surgeries cause BBB dysfunction which further leads to central nervous system syndromes (Yang et al. 2017, 2020). Thus, targeting the mechanisms to tighten the BBB could provide a novel strategy to inhibit the development of CNS complications associated with peripheral diseases. To better understand the effect of peripheral mediators and hormones on, and the interaction with the BBB, data mining of published data sets from various unbiased approaches could be a valuable strategy to identify novel players in BBB research. Methodological and technological advances will open new possibilities to study BBB function even in CNS diseases with subtle phenotypes, in which a vascular involvement is not obvious so far.

In conclusion, future research needs to tackle the mechanisms of communication between the periphery and the CNS in order to develop novel ways of adjuvant therapies for neurological symptoms, but also to better understand sex-specific function of drugs interfering with the hormonal system.

## Data Availability

No datasets were generated or analysed during the current study.
